# Changes in activation timing of knee and ankle extensors during gait are related to changes in heteronymous spinal pathways after stroke

**DOI:** 10.1186/1743-0003-11-148

**Published:** 2014-10-24

**Authors:** Joseph-Omer Dyer, Eric Maupas, Sibele de Andrade Melo, Daniel Bourbonnais, Sylvie Nadeau, Robert Forget

**Affiliations:** Centre de recherche interdisciplinaire en réadaptation, Institut de réadaptation Gingras-Lindsay de Montréal, Montréal, Canada; School of Rehabilitation, Faculty of Medicine, Université de Montréal, P.O. Box 6128, Montréal, Quebec Canada; UMT-Centre de Rééducation Fonctionnelle, Laboratoire de Physiologie de la Posture et du Mouvement PoM, Université Champollion, Albi - Université de, Toulouse, France

**Keywords:** Hemiparesis, Gait, Sensory afferents, Leg extensors, Spinal pathways, Propriospinal

## Abstract

**Background:**

Extensor synergy is often observed in the paretic leg of stroke patients. Extensor synergy consists of an abnormal stereotyped co-activation of the leg extensors as patients attempt to move. As a component of this synergy, the simultaneous activation of knee and ankle extensors in the paretic leg during stance often affects gait pattern after stroke. The mechanisms involved in extensor synergy are still unclear. The first objective of this study is to compare the co-activation of knee and ankle extensors during the stance phase of gait between stroke and healthy individuals. The second objective is to explore whether this co-activation is related to changes in heteronymous spinal modulations between quadriceps and soleus muscles on the paretic side in post-stroke individuals.

**Methods:**

Thirteen stroke patients and ten healthy individuals participated in gait and heteronymous spinal modulation evaluations. Co-activation was measured using peak EMG activation intervals (PAI) and co-activation amplitude indexes (CAI) between knee and ankle extensors during the stance phase of gait in both groups. The evaluation of heteronymous spinal modulations was performed on the paretic leg in stroke participants and on one leg in healthy participants. This evaluation involved assessing the early facilitation and later inhibition of soleus voluntary EMG induced by femoral nerve stimulation.

**Results:**

All PAI were lower and most CAI were higher on the paretic side of stroke participants compared with the co-activation indexes among control participants. CAI and PAI were moderately correlated with increased heteronymous facilitation of soleus on the paretic side in stroke individuals.

**Conclusions:**

Increased co-activation of knee and ankle extensors during gait is related to changes in intersegmental facilitative pathways linking quadriceps to soleus on the paretic side in stroke individuals. Malfunction of intersegmental pathways could contribute to abnormal timing of leg extensors during the stance phase of gait in hemiparetic individuals.

**Electronic supplementary material:**

The online version of this article (doi:10.1186/1743-0003-11-148) contains supplementary material, which is available to authorized users.

## Introduction

Following stroke, impaired coordination is frequently observed and manifests by the incapacity to activate muscles selectively[1]. This lack of voluntary control produces abnormal coupling of joint movements on the paretic side that can hamper motor task performance[1–3]. Altered motor coordination in the paretic leg among stroke patients is associated with functional deficits[4]. As a result of this lack of coordination, these patients often produce stereotypical co-activation of several muscles on the paretic side as they voluntarily attempt to move[1, 5]. These co-activations, which are commonly referred to as abnormal synergies, are defined as the simultaneous recruitment of muscles at multiple joints resulting in a stereotypical pattern of movement[6]. In the paretic leg of stroke patients, prevalent extensor synergy consisting of the co-contraction (i.e., co-activation) of the majority of the leg extensor muscles is often present throughout most of the stance phase of gait[7, 8]. This co-activation can be observed in EMG tracings showing the simultaneous activation of leg extensors during stance[6]. In the present study, the term “co-activation” will be used to describe the simultaneous EMG activity in knee and ankle extensor muscles[9]. This co-activation is a key component of extensor synergy[7] since it can produce abnormal coupling of knee and ankle extension, often resulting in an altered gait pattern after stroke[7, 10].

Since knee and ankle extensors are both anti-gravity muscles with out-of-phase activation during healthy gait, their abnormal co-activation could contribute to hemiparetic gait disabilities. The quadriceps muscle normally reaches its peak activation during the early stance phase in order to support body weight[11]. In turn, calf muscles demonstrate maximal activity during the late stance phase in order to control ankle dorsiflexion and produce push off[12]. In hemiparetic gait, prolonged activation of the quadriceps at the end of the stance phase[8, 13] may impede knee flexion in preparation for the swing phase. Premature activation of ankle extensors early in the stance phase[14, 15] could hamper body weight support upon initial foot contact[7]. These changes are consistent with abnormal co-activation of leg extensors on the paretic side during the stance phase of gait[14, 16].

Although the co-activation of leg extensors has been widely described in clinical literature, few studies have quantified its extent in the paretic leg during gait. The paucity of studies assessing muscular co-activation via EMG approaches may stem from limitations related to the normalization of EMG signals[17] and the determination of the timing of muscular activation[18], variables which allow inter-subject comparisons to be made. Analyses of EMG activity by factorization procedures have been used to objectively identify shared patterns of activation among different muscle groups in the paretic lower limb during gait[19, 20]. Through the use of a factorization procedure, it has been shown that the number of EMG modules required to describe muscle activation patterns in the paretic leg correlates with walking performance measures in post-stroke individuals[19].

Furthermore, the underlying mechanisms of leg extensor co-activation after stroke are not fully understood. Supraspinal and spinal mechanisms may both contribute to motor deficits in the paretic leg[21–23]. Spinal interneuronal systems are basic sensorimotor mechanisms that can integrate influences from sensory and descending pathways to modulate the activity of motoneurones (MNs)[9, 21]. Intersegmental or propriospinal pathways can regulate the activity of muscles acting at different joints[21, 24]. In humans, these pathways are assessed with electrophysiological methods, whereby conditioning stimulation is used to modulate the activity of a heteronymous muscle[25–27]. For example, intersegmental excitatory and inhibitory pathways linking quadriceps (Quads) to soleus (Sol) can be assessed by measuring the effects of femoral nerve (FN) stimulation on Sol activity[9, 21]. More precisely, FN stimulation induces early, short-term facilitation and later longer-lasting inhibition of both Sol H reflex and voluntary EMG, which have been attributed to projections from Quads to Sol group excitation and recurrent inhibition, respectively[28, 29]. An increase in early heteronymous facilitation and a decrease in later inhibition of Sol activity after FN stimulation have been found in stroke subjects[21]. Moreover, based on the results of this study, increased facilitation was correlated with level of motor coordination of the paretic leg[21]. This raises the question of whether co-activation of knee and ankle extensors in the paretic leg during gait is related to transmission changes in intersegmental pathways linking Quads to Sol. This study aims to (1) compare co-activation of knee and ankle extensors during gait between stroke and healthy individuals, (2) assess whether this co-activation is related to clinical measures of motor deficits after stroke, and (3) determine whether it is related to changes in heteronymous modulations of Sol voluntary EMG after FN stimulation in the paretic leg.

## Methods

### Participants

Thirteen stroke patients (mean ± SD: 51 ± 15 years; 5 females; 8 males) and ten healthy individuals (44 ± 13 years; 2 females; 8 males) of similar age (Mann-Whitney U: P = 0.24) participated in the study. The mean weight of the stroke participants (69.3 ± 9.2 kg) was not significantly different (P = 0.19) from that of the control participants (76.2 ± 11.9 kg). The mean height of the stroke participants (1.65 ± 0.07 m) was less (P = 0.02) than the mean height of the healthy participants (1.72 ± 0.06 m). All participants gave their written informed consent to participate in the study, which had been approved by the internal ethics committee for institutions affiliated with the Centre for Interdisciplinary Research in Rehabilitation of Greater Montreal (CRIR). Stroke participants were recruited based on the following criteria: a single cerebrovascular accident involving the motor cortex, internal capsulae or sub-cortical areas as documented by brain imagery findings and resulting in motor deficits of abrupt onset affecting the contralateral leg. All stroke participants were able to walk independently and continuously for at least 10 m, without a walking device (e.g., cane, orthosis) and had an activity tolerance of at least 2 hours including rest periods. Moreover, all stroke patients were able to perform the experimental task, which was to press on a fixed pedal with the forefoot in order to achieve and maintain a steady isometric calf muscle contraction for at least 5 s. All participants tested had detectable patellar and Achilles tendon reflexes at the paretic leg. Stroke subjects were excluded if they were on antispastic, anxiolytic or antidepressant medication at the time of the study, or if they had comprehensive aphasia, hemispatial neglect or a passive range of motion limitation at the paretic leg that could interfere with the experimental positioning. Moreover, participants did not have stimulators (e.g., pacemakers) or metallic implants and did not suffer from orthopaedic or neurological disorders other than stroke. Table 1 summarizes the demographic data for the stroke participants together with scores for clinical measurements of coordination, spasticity and motor recovery of the lower limb and gait speed, which were assessed as described below. With the exception of one participant (stroke individual # 13 in Table 1), all subjects had participated in a previous study at our laboratory[9]. Subjects were completely reassessed in terms of the needs of this study following a minimum delay of one week from completing their participation in the previous study[9].

**Table 1:** **Demographic and clinical data for stroke participants**

Participant	Age/gender	Side of brain lesion	Time since stroke (months)	CMSA at foot (/7)	LEMOCOT (# of times participant hit target)	CSI (/16)	Gait speed (m/s)
1	57/M	L	79	5	35	10	0.8
2	24/M	L	99	3	7	13	0.5
3	43/F	R	38	6	26	6	1.0
4	59/M	L	81	7	23	5	1.0
5	45/M	R	79	7	24	8	1.1
6	72/M	L	48	5	22	6	1.0
7	59/F	L	57	7	21	7	0.7
8	43/F	R	90	3	22	8	1.0
9	72/M	L	96	4	14	7	0.5
10	28/F	R	108	4	13	12	0.9
11	45/F	L	96	7	52	5	1.3
12	54/M	R	149	2	1	7	0.5
13	68/M	L	120	3	13	8	0.6

LEMOCOT: Lower Extremity Motor Coordination Test; CMSA at Foot: Chedoke-McMaster Stroke Assessment at the foot; CSI: Composite Spasticity Index.

### Clinical assessment

All clinical measurements were performed prior to any experimental procedures as described in the previous study[9]. Degree of spasticity of the paretic leg was measured using a reliable composite spasticity index (CSI) designed for stroke patients. Practical considerations on the use of a CSI are described by Levin and Hui-Chan (1993). Briefly, the CSI is a 16-point scale measuring the amplitude of an Achilles tendon tap (4 points), resistance to full-range passive ankle dorsiflexion at moderate speed (8 points), and duration of the clonus at the ankle (4 points). Interval values of 1-5, 6-9, 10-12 and 13-16 correspond to absent, mild, moderate and severe spasticity, respectively[30]. Motor coordination of the paretic leg was measured using the Lower Extremity Motor Coordination Test (LEMOCOT), validated among stroke individuals[4]. During this test, participants were seated and instructed to touch two standardized targets placed 30 cm apart on the floor with their foot, as fast and as accurately as possible during a 20-second period. The LEMOCOT score was calculated as the number of times the subject touched the two targets. Motor impairment was measured using the reliable Chedoke-McMaster Stroke Assessment (CMSA) subscale at the foot[31]. This subscale ranges from 1 (no residual motor function) to 7 (no residual motor impairment) and is based on Brunnstrom’s stages of motor recovery of the lower limb after stroke[5]. A self-selected comfortable walking speed over a 5-m distance without technical assistance (cane, walker or orthosis) was used as a standard and reliable method to measure gait performance in stroke participants[32, 33]. Average walking speed was measured during three trials. This clinical evaluation was followed by the experimental session, which was comprised of two assessments performed in random order that same day: 1) measurement of the co-activation of knee and ankle extensors during gait; and 2) electrophysiological evaluation of the heteronymous modulation of Sol by FN stimulation. Participants were not aware of the specific significance of the gait and electrophysiological assessments.

### Gait assessment

#### Experimental set-up and procedures

Three footswitches acting as force sensing resistors were placed under the shoe of each foot, at the heel, mid-sole and toes. ON and OFF signals from these switches were used to determine the supported and unsupported phases of the lower limbs. EMG activities of Sol, gastrocnemius lateralis (GL), rectus femoris (RF) and vastus lateralis (VL) were simultaneously recorded on both sides. The skin was first rubbed with alcohol to reduce its impedance. Disposable, self-adhesive, Ag/Ag-Cl surface electrodes (Ambu® Blue Sensor SP) were fixed in a bipolar configuration (at a 2-cm interelectrode distance with an 8 mm space between each recording areas) over the muscle bellies, based on SENIAM recommendations[34]. EMG signals were tested for crosstalk by performing standard muscle testing, rapid alternating movements and using a minimal interelectrode distance. EMG signals were collected during the gait assessment using a telemetric system (Telemyo 900, NORAXON Telemyo System, Scottsdale, AZ), relayed to a battery-powered amplifier (2000×) and transmitted to a receiver interfaced with a PC card. Signals were further digitalized at a sampling rate of 1200 Hz (bandwidth of 10 to 500 Hz) using software built on a LabVIEW 5.0 platform (National Instruments) and stored on a computer for subsequent analysis.

Stroke and healthy participants were instructed to walk on a 10-m walkway at a self-selected comfortable speed without technical assistance. Healthy participants were also asked to walk at a slower speed in order to match the self-selected comfortable speed of the stroke participants[35]. For each speed tested, participants were asked to complete three gait trials, with a minimum, 2-min rest period after each trial. Time was recorded for each trial using a digital stopwatch over a 5-m distance in the middle of the walkway. In this study, the comfortable self-selected gait speed of the stroke participants (mean ± SD: 0.85 ± 0.26 m/s) was lower (P <0.001) than the comfortable speed (1.22 ± 0.14 m/s) of the healthy participants but not different (Mann-Whitney U: P =0.62) from the self-selected slow speed (0.93 ± 0.14 m/s) of the healthy participants. Speed has been previously shown to influence muscular activation patterns and co-activation levels[36–38]. Therefore, all gait assessments and comparisons were performed at a comfortable speed in stroke participants and at a slow speed in healthy participants. At these speeds, the cadence of stroke participants (95 ± 18 steps/min.) was not different (P = 0.73) from the cadence of healthy participants (93 ± 10 steps/min.). Moreover, the step length on the paretic side (0.59 m ±0.13 m) was not different (P = 0.71) from the step length on the side evaluated in healthy participants (0.61 m ±0.05 m).

#### Gait assessment data analysis

All data analyses were performed off- line. Both sides were assessed in stroke participants and the side of control participants was randomly chosen. Foot switches and EMG signals were analyzed during each trial in the middle of the walkway, i.e., 2 m after the start line and 2 m before the finish line to avoid acceleration and deceleration bias. Three gait cycles were determined for each leg using the moment of heel strike on both sides. Each gait cycle selected was further subdivided into swing phase, stance phase, first (DS1) and second (DS2) double-support phases and single-support phase (SS) for each leg according to switch signals on both sides. Prior to analysis, all digital EMG signals were first filtered using a zero-phase shift fourth-order digital Butterworth band-pass filter (0-125 Hz) in order to reduce high frequency noise. For each gait cycle, digital EMG signals were full-wave rectified to obtain linear envelopes and were normalized to 100% of the entire gait cycle duration.

For each normalized gait cycle, the peak EMG activity achieved in each muscle was determined as the maximal EMG that can be measured in that muscle within a time window corresponding to 5% of the gait cycle during the stance phase. The timing of this peak during the gait cycle was expressed as a percentage of the length of the gait cycle. Two co-activation indexes were measured during the stance phase of each gait cycle between ankle and knee extensors: 1) peak activation intervals (PIA) related to the timing of co-activation, and 2) co-activation amplitude indexes (CAI) measuring relative EMG amplitudes of co-activation. PAI was determined by the time interval (expressed as a percentage of the length of the gait cycle) between peak activation of ankle extensors (Sol or GL) and knee extensors (VL or RF). For example, the PAI_Sol-VL_ represents the time interval between peak activation of Sol and VL during the stance phase. PAI has advantages over other measures since it is not dependent on the determination of EMG onset following an arbitrary threshold and it does not rely on unpredictable shapes of the estimated activation patterns[18]. Instead, it is based on the detection of peak muscular activation, which is easily determined and quantified objectively.

CAI was determined by mean EMG activation in a given muscle, expressed as a percentage of its maximal EMG during the stance phase, within the peak activation time window of a reference muscle at another joint. For example, CAI_Sol/Vlmax_ represents the mean level of Sol EMG during peak activation of VL. As for the PAI measure, CAI does not rely on the detection of EMG onset after an arbitrary threshold. Furthermore, CAI values were expressed as a percentage of maximal EMG activity of the muscle assessed during the gait cycle. This method of normalization was found to be more reliable for assessing levels of EMG activation during gait[39, 40] and more appropriate for inter-group comparisons[41, 42] than other normalization methods based on maximal contractions produced with a dynamometer[17, 41]. PAI and CAI presented in this study are the mean values of 9 gait cycles recorded during 3 gait trials.

### Assessment of the heteronymous modulation

The assessment of the heteronymous modulation presented in this section was performed as described in the previous study[9].

#### Experimental set-up and instrumentation

**Figure 1:**
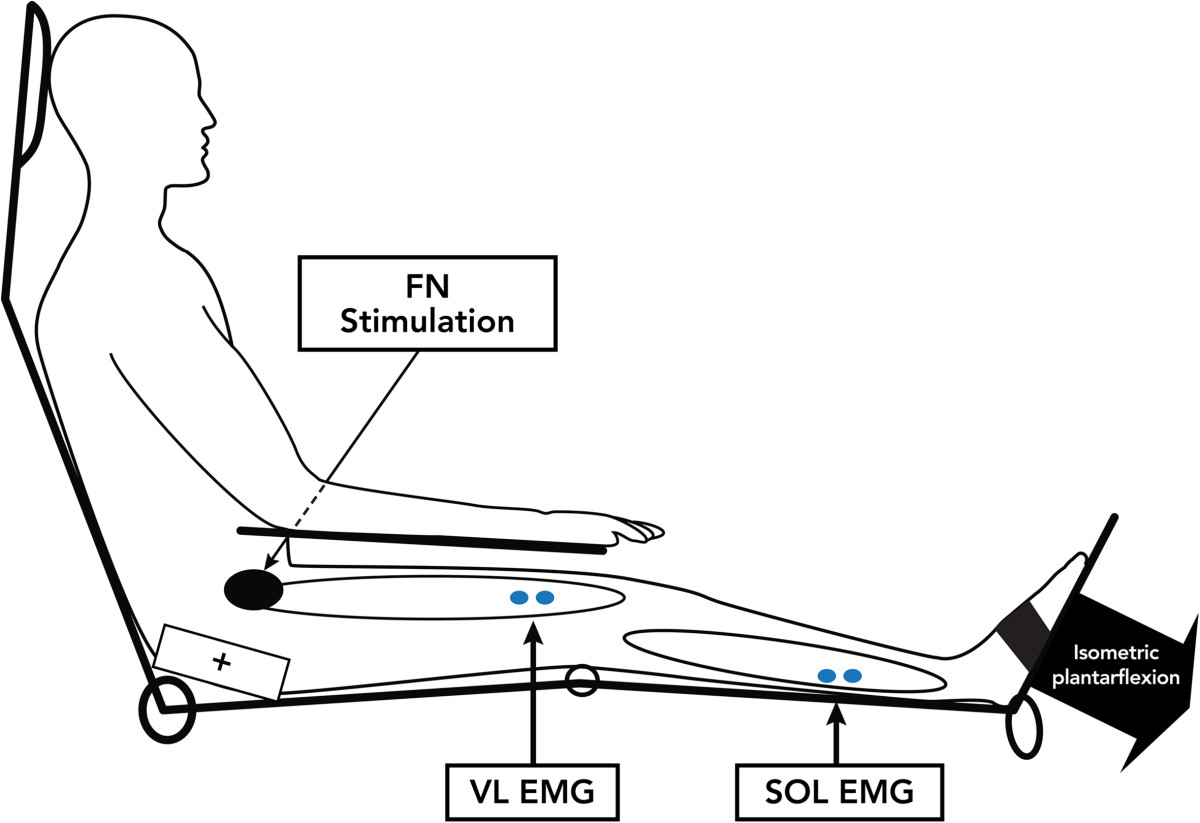
**Participants were seated with the hip flexed (80°), the knee slightly flexed (10°), the ankle slightly plantarflexed** **(110°)** **and the foot on a fixed platform on the tested side.** Participants produced isometric plantarflexions. EMG activities at Sol (SOL EMG) and VL (VL EMG) were recorded. The FN was stimulated with a cathode at the femoral triangle and an anode on the postero-lateral aspect of the buttock (FN stimulation).

Participants were seated comfortably in an adjustable reclining armchair with the foot strapped to a fixed pedal. The paretic leg was tested in stroke patients and the side tested in control individuals was randomly selected. The leg tested was positioned with the hip flexed (80°), the knee slightly flexed (10°) and the ankle slightly plantarflexed (110°) (see Figure 1). The FN was stimulated with a 1-ms duration monophasic rectangular pulse (Grass S88 stimulator) delivered by a cathode (2-cm-diameter half-ball) at the femoral triangle and an anode (11.5 cm × 8 cm) placed at the postero-lateral aspect of the buttock. The intensity of the stimulation was progressively increased to determine the thresholds of the H reflex and of the M response (MT) for VL. The intensity was then maintained at 2 × MT for the remainder of the experiment. EMG activities of Sol and VL were recorded (Grass, model 12 acquisition system) using bipolar surface electrodes (*Beckmann*, Ag-AgCl; 9 mm diameter) placed 2 cm apart (center-to-center). The recording electrodes were secured over the belly of VL (on the distal third of the antero-lateral aspect of the thigh) and Sol (on the distal third of the postero-lateral aspect of the leg, just below the lateral gastrocnemius). EMG signals were first amplified (5000×), then filtered (30–1000 Hz) (*Grass*, model 12 A 5) and finally, digitalized at a sampling rate of 5 kHz. EMG signals were displayed on an oscilloscope and stored on computer for off-line analysis[9].

#### Experimental protocol for modulation assessment

Participants were instructed to press on the fixed platform with the forefoot in order to produce isometric plantarflexion contractions. The level of EMG activity of Sol during maximal isometric voluntary contractions during 5 s of plantarflexion (EMG_max_) was first determined for each participant (mean of three trials). All participants then had to produce sustained isometric plantarflexion contractions to activate Sol at 30% of its EMG_max_. Throughout the experiment, an analogue voltmeter facing the participant displayed visual feedback on the level of voluntary activity achieved at Sol (rectified and integrated EMG activity surface) for baseline control. Contractions had to be maintained for at least 3 s and a minimum rest period of 20 s was allowed between each trial. Random FN stimulations (2 × MT) were performed during these contractions so that the stimulation occurred in about one out of three contractions. The interval between the onset of Sol activation and the stimulation was also randomized. This ensured that participants would not be able to predict during which contraction the stimulation would be applied, or exactly when it would occur after the onset of Sol activation. For each leg tested, unconditioned and conditioned voluntary EMG activity values for 10 FN stimulation trials were recorded during Sol voluntary contractions[9].

#### Modulation data analysis

Assessments of the heteronymous modulation were all performed off-line. For each trial, Sol EMG was full-wave rectified from 100 ms before to 80 ms after FN stimulation. Latency of changes in Sol EMG were expressed in terms of zero central delay, i.e., when the fastest FN Ia volley was expected to arrive at the segmental level of the soleus motoneuron (MN) pool. This zero central delay was calculated for each participant based on latency Sol H reflex and the difference in afferent conduction times between homonymous and heteronymous Ia pathways for each leg tested[29, 43]. Early facilitation was found to peak within 6 ms after zero central delay in both healthy and stroke participants tested in this study. Early facilitation reached a maximal duration of 12 ms in healthy controls and 36 ms in some severely affected stroke participants. Later inhibition can be observed in healthy participants as early as 6 ms after zero central delay and can last up to 40 ms. Thus, the level of facilitation for each participant was measured by the surface of Sol EMG within the window of analysis from 0 to 6 ms after the zero central delay (about 25 to 31 ms after FN stimulation). The later modulation was assessed within three, consecutive, 12-ms time windows of analysis, from 12 to 24 ms, 24 to 36 ms and 36 to 48 ms after the zero central delay (about 37 to 73 ms after FN stimulation). Facilitation and inhibition levels were measured at each time window during each trial as the difference between the integrated rectified EMG after the conditioning stimulation (conditioned EMG) and before the stimulation (unconditioned EMG). This difference was expressed as a percentage of the control EMG measured within a 100-ms period of time just before stimulation, and was then normalized for the duration of the time windows of analysis for facilitation (6 ms) and later modulation (12 ms) to allow comparisons to be made. Mean modulation of Sol voluntary EMG was assessed during ten isometric contraction trials, for each leg tested[9].

### Statistics

Mann-Whitney U and Wilcoxon tests were used during the gait assessment to compare co-activation levels and spatio-temporal characteristics of the gait cycle between and within groups, respectively. Spearman rank correlations were used to correlate clinical scores of coordination (LEMOCOT), motor recovery (CMSA), spasticity (CSI) and gait speed with levels of co-activation measured in stroke individuals.

For the assessment of the heteronymous modulation, an analysis of variance (ANOVA) comparison using Scheffe’s method was performed to determine whether or not there was significant facilitation and inhibition throughout the assessment period before and after FN stimulation. Mann-Whitney U and Wilcoxon tests were used to compare levels of modulations between and within groups, respectively. Pearson correlations were used to correlate levels of co-activation with levels of heteronymous modulation. P values ≤0.05 were considered significant. All statistical analyses were performed using the Statistical Package for Social Science (SPSS) software, version 19 for Windows.

## Results

### Gait characteristics across participants

**Figure 2:**
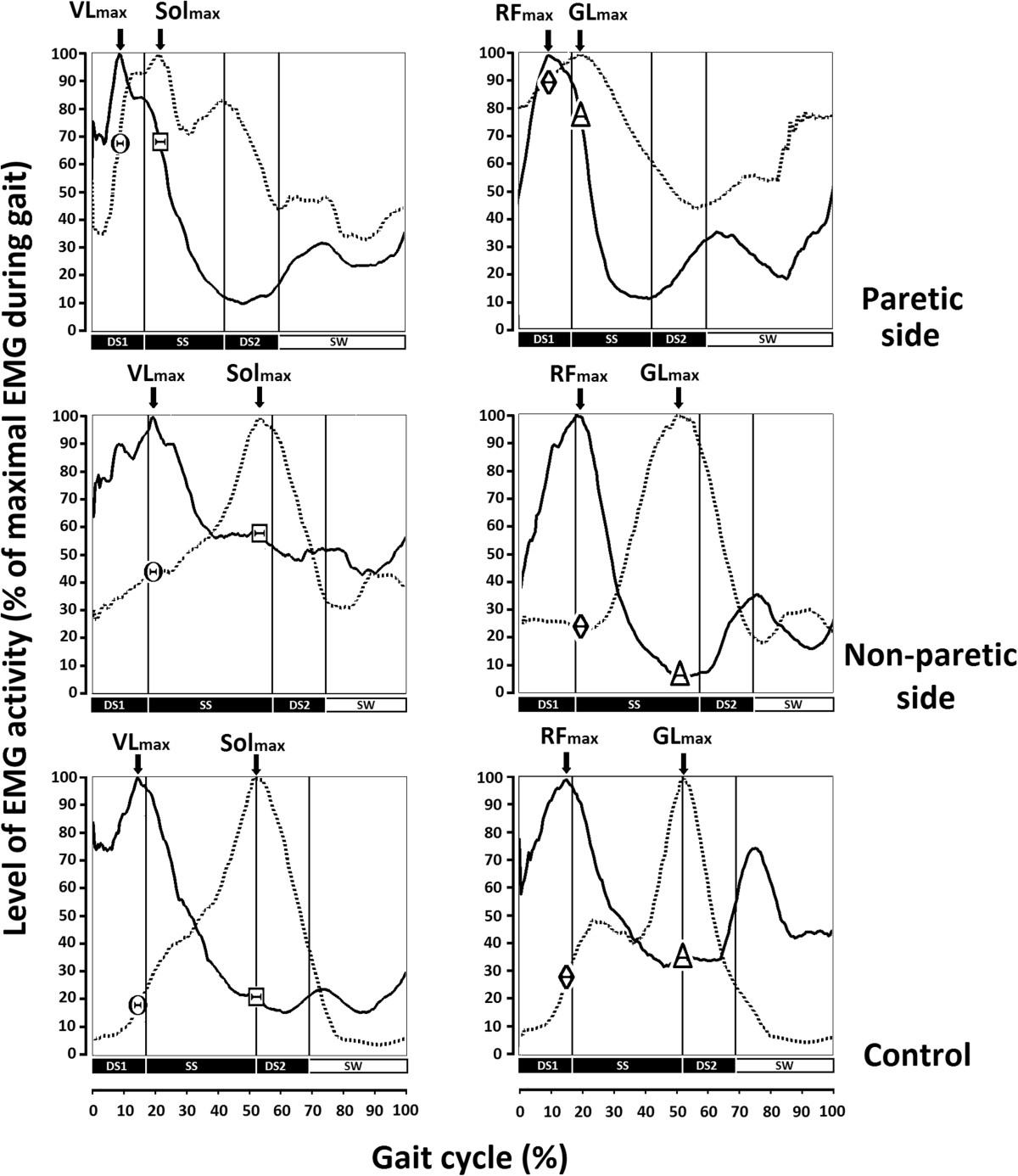
**Mean rectified EMG of vastus lateralis,** **soleus,** **rectus femoris and gastrocnemius lateralis during gait in a stroke participant and a healthy participant.** Tracing of averaged rectified EMG activities of knee extensors (continuous line) and ankle extensors (dotted line) are presented for the paretic (upper panel) and non-paretic (middle panel) sides of a stroke participant (# 4 in Table 1) and a healthy participant (lower panel) walking at 0.99 m/s and 1.04 m/s, respectively. Tracing are averaged for 9 cycles, expressed as a percentage of maximal EMG. The duration (length of time) of the first double-support (DS1), single-support (SS), second double-support (DS2) sub-phases of stance and of the swing phase (SW) are presented in relation to the entire gait cycle. Co-activation of vastus lateralis (VL) and soleus (Sol) is presented in the left panel. Co-activation of rectus femoris (RF) and gastrocnemius lateralis (GL) is presented in the right panel. Arrows represent the latencies in maximal activation of vastus lateralis (VL_max_), soleus (Sol_max_) rectus femoris (RF_max_) and gastrocnemius lateralis (GL_max_). Circles represent the amplitude of the soleus co-activation index during peak activation of VL (CAI_Sol/Vlmax_). Squares represent the amplitude of vastus lateralis co-activation during peak activation of Sol (CAI_VL/Solmax_). Lozenges represent the amplitude of gastrocnemius lateralis co-activation during peak activation of RF (CAI _GL/RFmax_). Triangles represent the amplitude of rectus femoris co-activation during peak activation of GL (CAI _RF/GLmax_).

Spatio-temporal characteristics and co-activation levels during gait were modified in stroke participants. Mean EMG activities of VL, RF, Sol and RF during the gait cycle (average of 9 strides) are presented for the paretic and non-paretic legs of one stroke participant (# 4 in Table 1) and for one leg of a healthy participant (see Figure 2). The mean duration of the stance phase (DS1 + SS + DS2) on the paretic side (mean of 9 strides ± SEM: 59 ± 0.4% of the gait cycle) was shorter (P <0.01) than that on the non-paretic side for the stroke participant (76 ± 0.4%), and the control participant (69 ± 1.4%) (see Figure 2). The peak activation interval between VL_max_ and Sol_max_ (PAI _VL-Sol_) on the paretic side (mean of 9 strides ± SEM: 12 ± 5% duration of the gait cycle) was shorter (P <0.05) than the PAI _VL-Sol_ on the non-paretic side for the stroke participant (34 ± 3%) and the control participant (38 ± 1%) (see left panel in Figure 2). The co-activation amplitude index of Sol at the peak activation of VL (CAI_Sol/Vlmax_) (see Θ in Figure 2) on the paretic side (mean of 9 strides ± SEM: 68 ± 5% of Sol EMG max) was higher than the CAI_Sol/Vlmax_ on the non-paretic side of stroke participants (44 ± 7%) and higher than that in the control participants (18 ± 2%). Similarly, the co-activation amplitude index of VL during peak activation of Sol (CAI_VL/Solmax_) (see Ξ in Figure 2) on the paretic side (69 ± 9% of VL EMG max) was not significantly different from the value observed on the non-paretic side (58 ± 3%) but was higher (P < 0.01) than found in the control participants (22 ± 0.3%).

The peak activation interval between RF_max_ and GL_max_ (PAI_RF-GL_) on the paretic side (8 ± 2% duration of the gait cycle) of stroke participants was shorter (P <0.05) than the PAI _RF-GL_ on the non-paretic side (30 ± 4%) and shorter than that observed in control participants (37 ± 1%) (see right panel Figure 2). The co-activation amplitude index of GL during peak activation of RF (CAI_GL/RFmax_) (see ◊ in Figure 2) on the paretic side (88 ± 7% of GL EMG max) was higher than the CAI_GLRFmax_ on the non-paretic side (23 ± 5%) and higher than that found among the control participants (27 ± 3%). Similarly, the co-activation amplitude index of RF when GL was at its peak activation (CAI_RF/GLmax_) (see Δ in Figure 2) on the paretic side (75 ± 8% of RF EMG max) was significantly different (P <0.05) from the value observed on the non-paretic side (6 ± 2%) as well as that observed in the control participant (33 ± 1%).

### Gait assessment across groups

**Figure 3:**
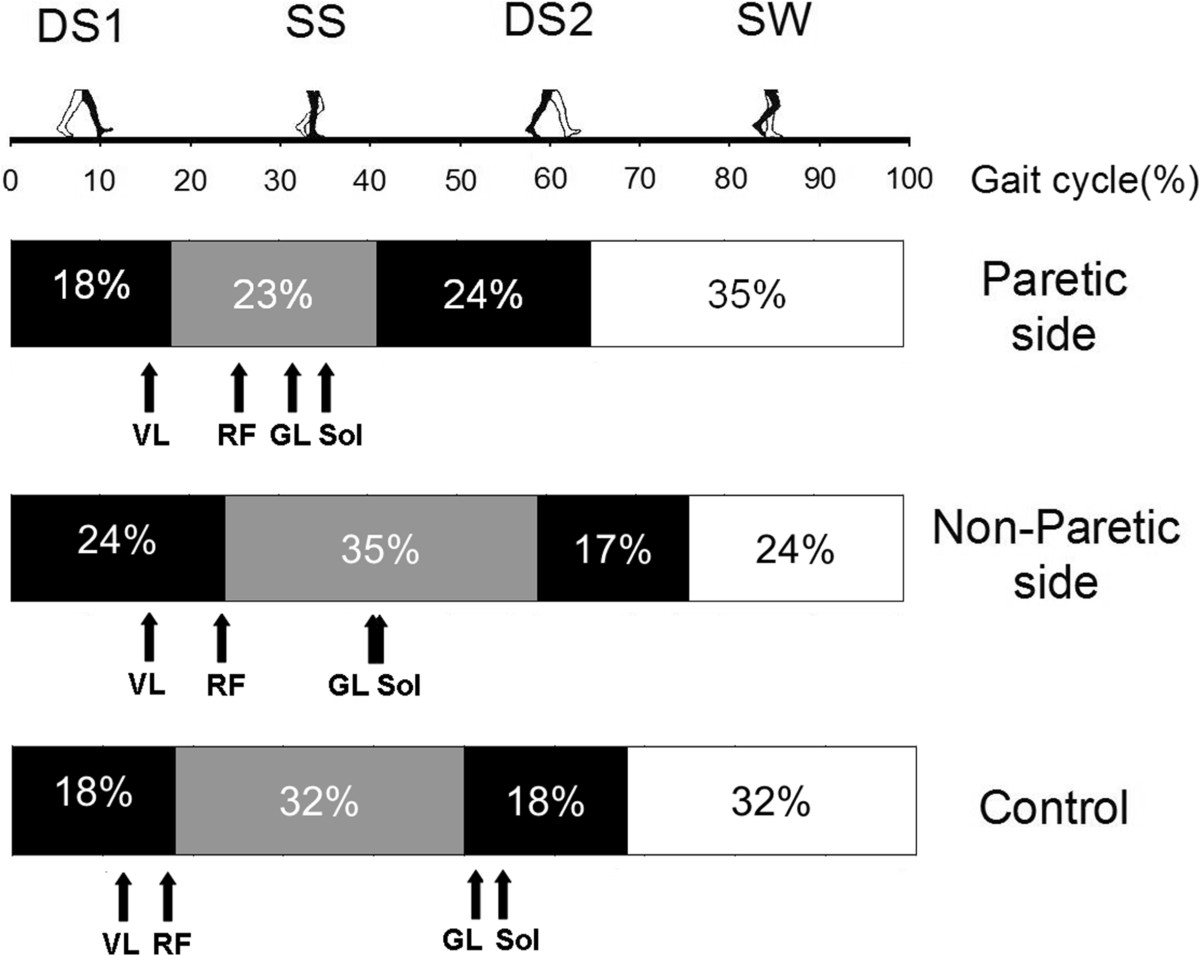
**Group comparisons of gait cycles in 13 stroke and 10 healthy participants.** The mean duration of gait cycle phases are presented for the paretic (upper bar) and non-paretic (middle bar) sides of stroke participants and for control participants (lower bar). Percentages of first double-support (DS1), single-support (SS), second double-support (DS2) sub-phases of the stance phase and of the swing phase (SW) are expressed as a percentage of the duration of the gait cycle. The schematic representation of the position of the lower limbs during each phase is presented with the black leg being the one assessed. Arrows represent the mean latencies in the peak activation of vastus lateralis (VL), rectus femoris (RF), gastrocnemius lateralis (GL) and soleus (Sol) during the gait cycle.

**Figure 4:**
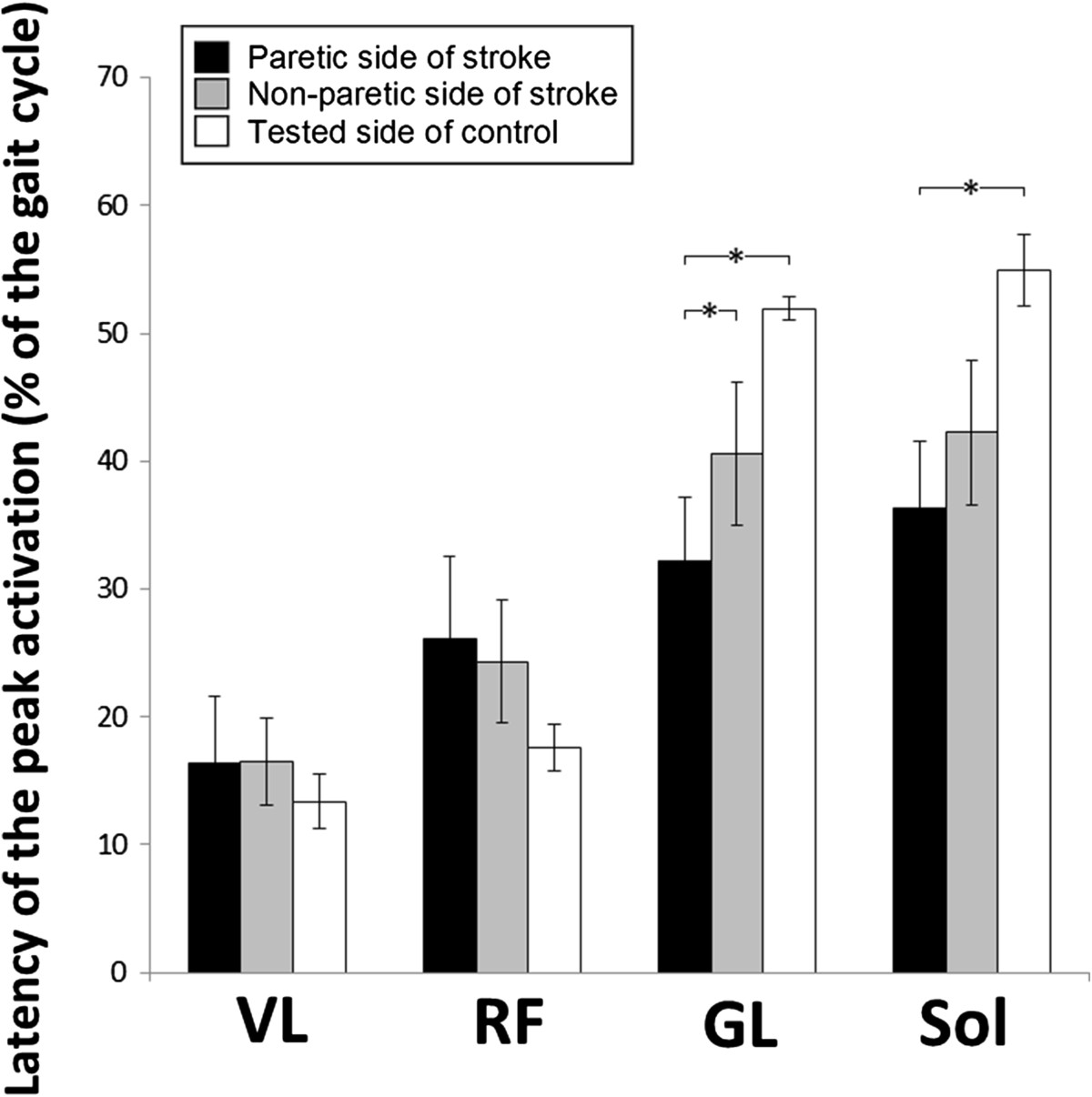
**Group comparisons of peak activation latencies for vastus lateralis**, **rectus femoris**, **gastrocnemius lateralis and soleus during gait in 13 stroke and 10 healthy participants.** Peak activation latencies for vastus lateralis (VL), rectus femoris (RF), gastrocnemius lateralis (GL) and soleus (Sol) during the stance phase of gait are expressed as a percentage of the gait cycle. Mean peak activation latencies are presented for the paretic (black bars) and non-paretic (grey bars) sides of stroke participants and control participants (white bars). Vertical bars =1 SEM. Asterisks represent significant differences between groups (* p ≤0.05).

Relative components of gait cycle sub-phases were modified on the paretic side of stroke participants (see Figure 3). The mean duration of the stance phase (DS1 + SS + DS2) on the paretic side (mean ± SD: 65 ± 4% of the duration of the gait cycle) was shorter (P < 0.05) than the duration on the non-paretic side of stroke participants (76 ± 6%) and shorter than that in control participants (68 ± 3%) (see Figure 3).Timing of peak activation of ankle extensors was modified in the paretic leg of stroke participants (see Figure 4). The same was not true for knee extensors. Mean latencies in peak activation of VL and RF on the paretic side were not significantly different from the values on the non-paretic side in stroke participants and in control participants. The mean latency in peak activation of GL on the paretic side was shorter (P < 0.05) than that on the non-paretic side and that in control participants. The latency in peak activation of Sol on the paretic side was not significantly different from that on the non-paretic but was shorter in control participants. Moreover, peak activation latencies of VL, RF, GL and Sol on the non-paretic side of stroke individuals were not different from the values observed in control participants.

**Figure 5:**
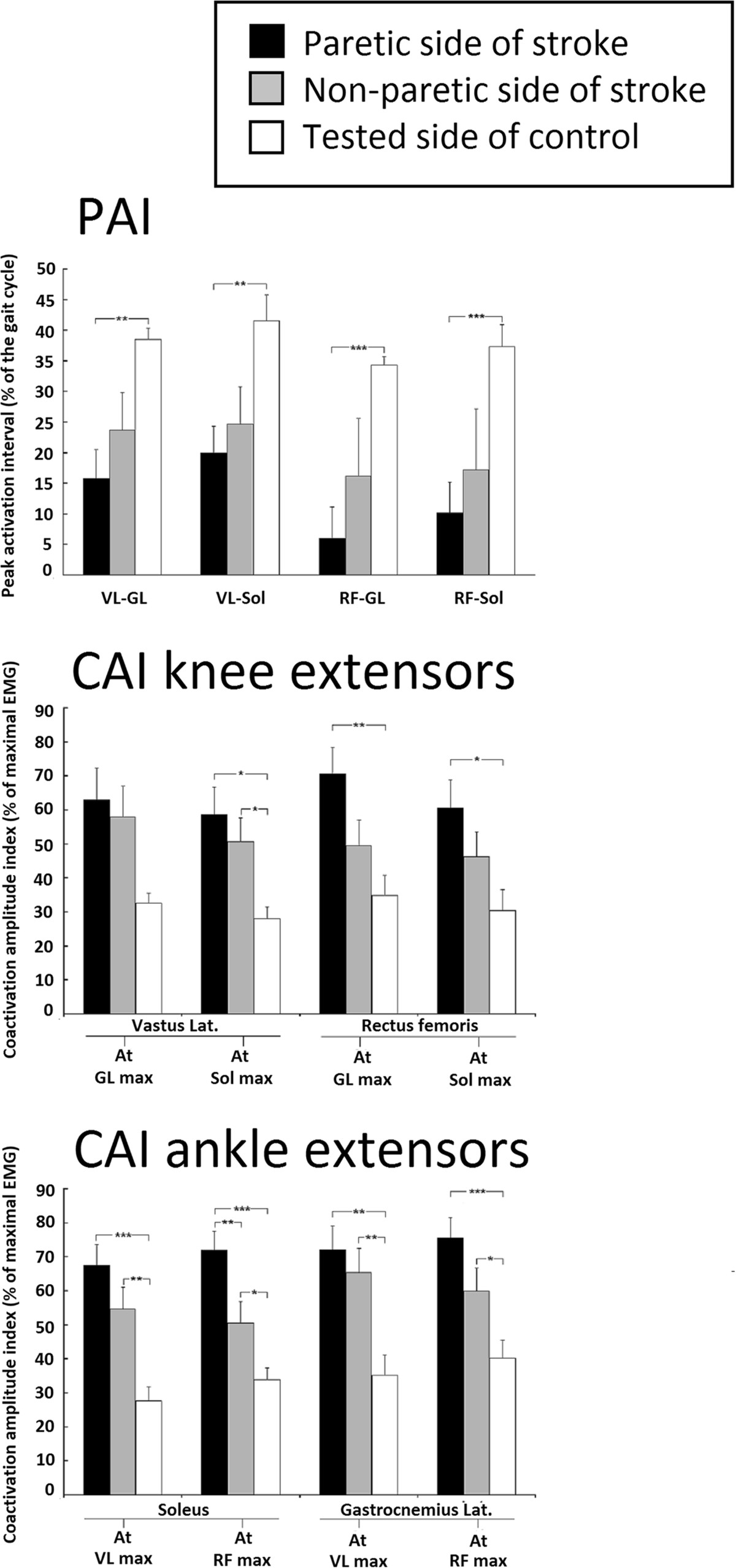
**Group comparisons of co-** **activation indexes in 13 stroke and 10 healthy participants.** Mean peak activation intervals (PAI) and co-activation amplitude indexes (CAI) measured during the stance phase are presented for the paretic (black bars) and non-paretic (grey bars) sides of stroke participants and for control participants (white bars). PAIs between knee extensors (vastus lateralis: VL or rectus femoris: RF) and ankle extensors (soleus: Sol and gastrocnemius lateralis: GL) are expressed as a percentage of the gait cycle duration (upper panel). CAI of knee extensors (VL and RF), expressed as a percentage of their maximal EMG activation during peak activation of ankle extensors (at Sol_max_ and GL_max_), are also presented (middle panel). CAI of ankle extensors (Sol and GL), expressed as a percentage of their maximal EMG activation, during peak activation of knee extensors (at VL_max_ and RF_max_) are presented (lower panel). Vertical bars =1 SEM. Asterisks represent significant differences between groups (* p ≤0.05; ** p ≤0.01; *** p ≤0.001).

Co-activation of knee and ankle extensors during the stance phase in stroke participants was higher than in control participants (see Figure 5). Mean peak activation intervals (PAI) between knee and ankle extensors on the paretic side of stroke participants were shorter than the PAIs in the control participants but were not significantly different from the values on the non-paretic side. Co-activation amplitude indexes (CAI) of knee extensors during peak activation of ankle extensors were significantly higher on the paretic side of stroke participants than the CAI of knee extensors in control participants, except for the level of VL activation at GL_max_ (CAI_VL/Glmax_). These CAIs were not significantly different between the paretic and non-paretic side. All ankle extensor CAIs during peak activation of knee extensors measured on the paretic and non-paretic sides of stroke participants were greater than the values recorded for control participants.

PAI _VL-Sol_ correlated with levels of spasticity (CSI: Spearman r = -0.65; P =0.016) and motor impairment (CMSA: r =0.55; P =0.05) of the paretic leg. CAI_VL/Solmax_ correlated with levels of spasticity (CSI: r =0.66; P =0.015), motor impairment (CMSA: r = -0.66; P =0.014) and coordination (LEMOCOT: r = -0.69; P =0.009). CAIs of ankle extensors during maximal quadriceps activation did not correlate with clinical measures.

### Heteronymous modulation across participants

**Figure 6:**
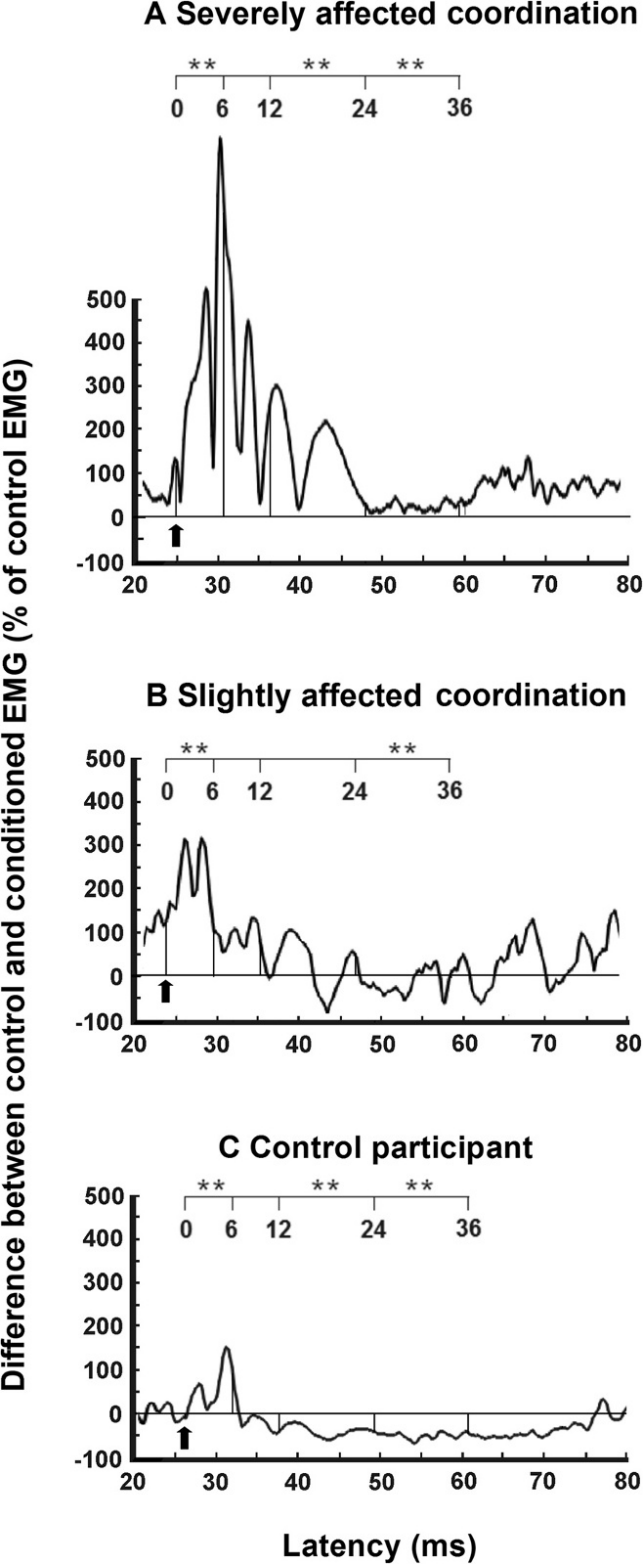
**Effects of femoral nerve stimulation on voluntary soleus EMG activity in two stroke participants and one control participant.** Tracings of averaged rectified EMG activities of ten trials are presented for the paretic sides of stroke participants with severely **(A)** or slightly **(B)** impaired coordination and for the right leg of a control participant **(C)**. Arrows indicate the zero central delay, which is the expected time of arrival of the fastest FN Ia volley at the segmental level of the soleus motoneuron (Mn) pool. The latency scale is presented from 20 ms to 80 ms after FN stimulation (lower scale) and from 0 to 36 ms after the zero central delay (upper scale). Horizontal lines represent the mean amplitude of the unconditioned EMG activity (baseline EMG level). Facilitation was assessed within the time window from 0 to 6 ms after the zero central delay. Asterisks represent significant modulations of soleus within three time windows of analysis from 0 to 6 ms, 12 to 24 ms and 24 to 36 ms after the zero central delay (* p ≤0.05; ** p ≤0.01; *** p ≤0.001).

An increase in early facilitation and a decrease in the later inhibition of Sol voluntary EMG induced by FN stimulation was observed in the paretic leg of stroke participants. The heteronymous modulation of Sol EMG is presented in stroke participants with severely (participant #10 in Table 1) and mildly (participant # 11 in Table 1) impaired coordination (LEMOCOT score: 13 and 52, respectively) and in a control participant (see Figure 6). In the first time window of analysis from 0 to 6 ms after zero central delay, early facilitation of the severely affected stroke participant (mean ± SEM; increase of 397 ± 24% of Sol control EMG surface) was higher (P <0.01) than facilitation in the slightly impaired participant (218 ± 7%) and in the control participant (76 ± 6%). From 12 to 24 ms after the zero central delay, the facilitative modulation observed in the severely impaired participant (157 ± 15%) was higher than (P <0.05) the modulation observed in the mildly impaired stroke participant (16 ± 7%) and significantly different (P < 0.001) from the inhibition (decrease of 51 ± 4% of Sol control EMG surface) observed in the control participant. In the next time window from 24 to 36 ms, the facilitation observed in the severely impaired participant (increase of 30 ± 9%) was significantly different (P <0.05) from the inhibition observed in the mildly impaired stroke participant (decrease of 27 ± 4%) and significantly different from the inhibition (decrease of 59 ± 1%) observed in the control participant.

### Heteronymous modulation across groups

**Figure 7:**
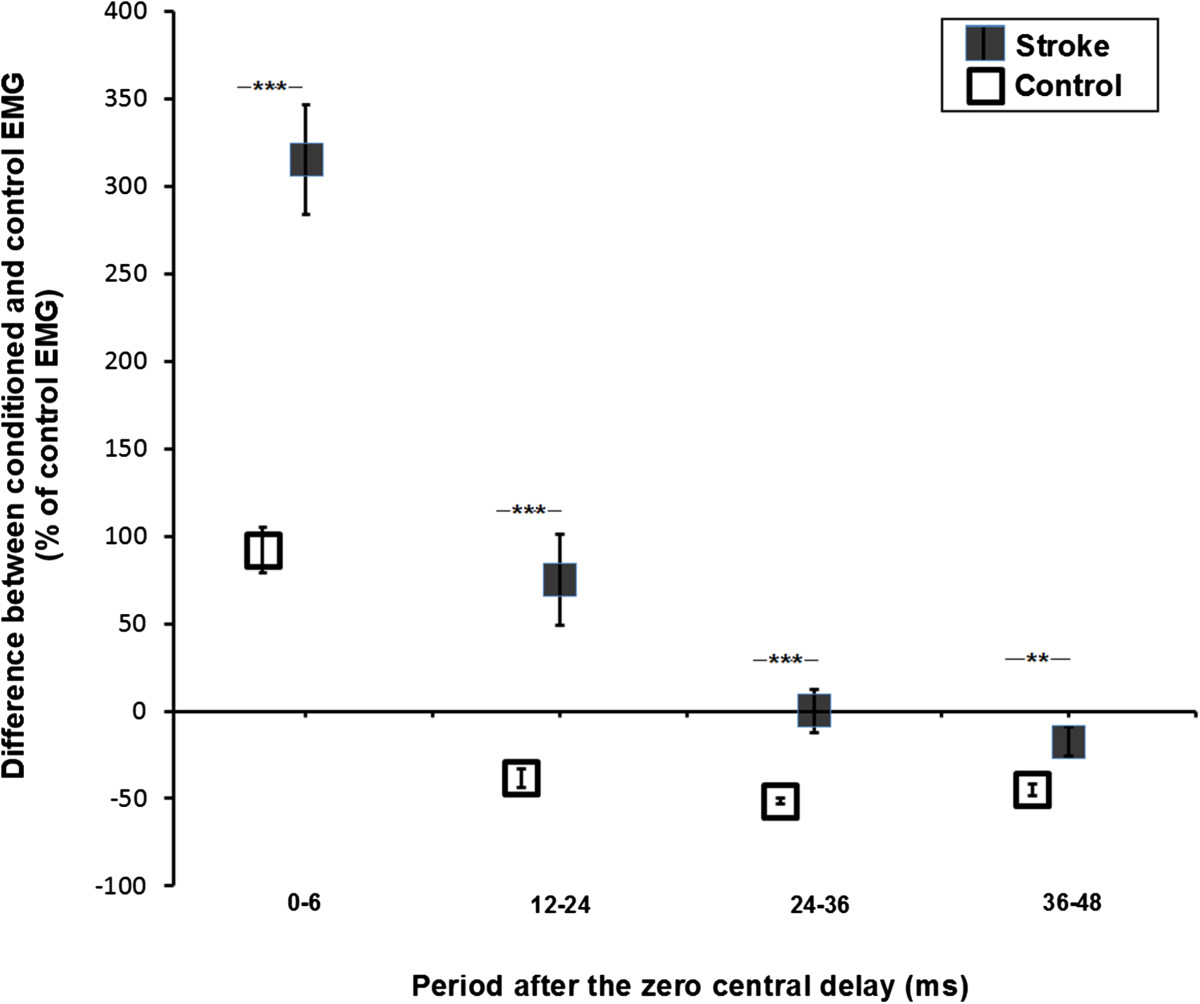
**Effects of femoral nerve stimulation on soleus voluntary EMG activity in 13 stroke and 10 healthy participants.** Mean modulation of soleus voluntary EMG activity induced by FN stimulation for the stroke group (black squares) and the control group (white squares) expressed as a percentage of unconditioned EMG. Modulations are presented within the four time windows from 0 to 6 ms, 12 to 24 ms, 24 to 36 ms and 36 to 48 ms after the zero of central delay. Positive values (i.e., above zero on the ordinate scale) are facilitation and negative values are inhibition. Vertical bars =1 SEM. Asterisks represent significant differences in modulation between the control and stroke participants (* p ≤0.05; ** p ≤0.01; *** p ≤0.001).

The mean heteronymous modulation of Sol voluntary EMG induced by FN stimulation observed in the stroke group was different from that of the control group (see Figure 7). The early facilitation (from 0 to 6 ms after the zero of central delay) observed in the stroke group was greater than the facilitation in the control group. In the next three time windows, the modulation observed in the stroke group was different (P <0.01) compared with the inhibition observed in the control group.

### Correlations between co-activations and heteronymous modulations

Correlations were found between co-activation indexes and changes in heteronymous modulations in stroke participants but not in healthy participants. Table 2 presents the correlations found between co-activation indexes assessed during gait and the heteronymous modulation of Sol measured during the four consecutive time windows of analysis after FN stimulation in both groups. The peak activation interval between VL and ankle extensors (PAI_VL-Sol_ and PAI_VL-GL_) and the level of co-activation of VL during peak activation of Sol (CAI_VL/Solmax_) correlated with the heteronymous modulation within the first time window from 0 to 6 ms after the zero central delay in stroke participants. The PAI_VL-Sol_ was consistent with the modulation within the subsequent time window from 12 to 24 ms. PAI_VL-GL_ correlated with the modulation from 24 to 36 ms. No significant correlations were found between the co-activation indexes and heteronymous modulations in the control participants.

**Table 2:** **Correlation coefficients** (**Pearson**) **between heteronymous modulations produced by FN stimulation and co**-**activation indexes during gait**

Group	Modulation	Co-activation indexes							
	Time window (ms after ZCD)	PAI_VL-Sol_	PAI_VL-GL_	PAI_RF-Sol_	PAI_RF-GL_	CAI_VL/at Solmax_	CAI_Sol/at VLmax_	CAI_RF/at GLmax_	CAI_GL/at RFmax_
**Stroke**	(0-6)	-0.73**	-0.62*	-0.41	-0.34	0.57*	-0.32	-0.44	-0.47
	(12-24)	-0.65*	-0.45	-0.24	-0.09	-0.43	-0.23	-0.22	-0.24
	(24-36)	-0.550	-0.62*	-0.27	-0.35	-0.49	-0.17	-0.26	-0.38
	(36-48)	-0.21	-0.40	-0.14	-0.32	-0.29	-0.01	-0.08	-0.22
**Control**	(0-6)	-0.40	-0.55	-0.29	-0.24	-0.14	-0.33	-0.16	-0.29
	(12-24)	-0.04	-0.33	-0.45	-0.55	-0.10	-0.28	-0.14	-0.25
	(24-36)	-0.24	-0.11	-0.28	--0.14-	-0.05	-0.33	-0.16	-0.16
	(36-48)	-0.10	-0.11	-0.12	-0.03	-0.10	-0.11	-0.13	-0.11

PAI: peak activation interval; CAI: co-activation amplitude index; Sol: soleus; GL: gastrocnemius lateralis; VL: vastus lateralis and RF: rectus femoris; ZCD: zero central delay; Significant correlations are in bold; *p ≤ 0.05; **p ≤ 0.01.

## Discussion

### Changes in gait cycle and muscle co-activation after stroke

Our results show an increased co-activation of knee and ankle extensors in hemiparetic gait compared to healthy participants. The peak activation interval (PAI) and co-activation amplitude index (CAI) measures used in the present study were sensitive enough to detect abnormal levels of EMG co-activation of leg extensors during gait in the paretic and non-paretic legs of stroke individuals. Other studies have quantified increased co-activation of leg extensors on the paretic side during static contractions. Abnormal torque coupling between hip adduction and knee extension has been reported during submaximal static contractions while standing with the leg positioned in the toe-off position of gait[44]. An abnormal increase in co-activation of hip and knee extensors has been reported during maximal isometric hip and knee extensions on the paretic side while standing[45]. Shared patterns of activation among different muscle groups have been identified in the paretic lower limb during gait using EMG data analyses based on factorization procedures[19, 20]. Accordingly, a shared pattern of activation was observed between ankle extensors and knee extensors (RF and VM) throughout the stance phase of the paretic leg but not among controls[20].

All PAIs measured in the present study were at least two times shorter on the paretic side compared to those measured in the control group. PAI values on the non-paretic side also tended to be shorter than the values recorded for control participants. Lower PAI values are essentially attributable to the earlier activation and rapid increase in ankle extensor activity which leads to increased temporal co-activation of leg extensors in hemiparetic gait. CAI values in stroke participants were also twice higher than values in healthy participants. This is consistent with a study on standing position that showed co-activation of knee and ankle extensors during maximal static ankle and knee extensions on the paretic side, with recorded values twice those of the healthy controls[2]. Furthermore, the amplitude of the co-activation measured during gait in the present study was higher than the levels measured in a standing position under static conditions in stroke and healthy participants[2]. This suggests that the co-activation of leg extensors on the paretic side might be higher during a dynamic task such as gait than during a static task.

It is important to consider that the co-activation measures used in the present study are not independent. In fact, a reduction in the peak activation interval (PAI) should coincide with an increase in co-activation amplitude index (CAI). Both measures of co-activation used in the present study were closely related to the timing of knee and ankle extensor activation during the gait cycle. This timing is essentially measured by the PAI. However, the PAI does not take into account the relative variation in EMG amplitude during the gait cycle. Such modulation of EMG amplitude throughout the gait cycle can also be referred as the shape of the EMG profile for each muscle. The aim of the present study was to compare the co-activation of knee and ankle extensors between stroke and control participants. Therefore, one should consider potential differences in the shape of the EMG tracings of knee and ankle extensors between stroke and control participants. Some characteristics of the EMG recordings, such as the modulation of EMG amplitude throughout the gait cycle and the EMG ascending time to reach peak activation, are expected to be different in stroke and control participants. In this respect, CAI provides complementary information based on PAI since CAI measure takes into account the relative EMG amplitude of the muscle at a specific moment within the gait cycle. This may explain why co-activation differences between the non-paretic leg of stroke participants and the tested leg in controls have been found with CAI measures but not with PAI measures. Future studies should investigate whether PAI and CAI correlate with measures of co-activation based on more complex mathematical analyses of EMG signals such as factorization analyses.

One limitation of the present study is that only knee and ankle extensors have been assessed. Therefore, this study did not determine whether knee and ankle extensors co-activate with other muscles or whether co-activations of other muscles are greater than that of knee and ankle extensors. Stroke can lead to reduced control of many lower limb muscles during gait. Earlier activation of ankle extensors in hemiparetic gait might be triggered by their co-activation with muscles other than the knee extensors which are activated during mid-stance. The present study focused specifically on the assessment of knee and ankle extensors in an attempt to quantify the pathological extensor synergy often described in hemiparetic gait.

### Possible mechanisms behind inter-joint co-activation

The mechanisms underlying global synergistic activation of the leg extensor muscles when stroke patients attempt to move the paretic leg are not fully understood. Weakness, changes in motor unit recruitment, changes in supraspinal influences and dysfunction of spinal pathways have been suggested as possible mechanisms involved in abnormal synergistic co-activations after stroke[9, 46]. In terms of weakness, some evidence suggests that abnormal inter-joint co-activation is an adaptive strategy to compensate for unequal distribution of weakness across joints and muscles in the paretic limb[46]. Conversely, other studies propose that the presence of co-activation may impede torque generation and contribute to weakness rather than it being the result of lost strength in the paretic leg[45, 47]. The inability to adequately recruit motor units, albeit more associated with weakness, could indirectly contribute to the presence of co-activation in the paretic limb. A reduction in the frequency of motoneurone discharges and in the number of motor units available[48–50] could play a role in limited movement repertoire and therefore synergistic co-activation in hemiparesis.

Changes in supraspinal influences could affect the ability to activate muscles selectively. For example, the neural reorganization resulting from stroke is associated with an enlargement of the cortical areas activated during voluntary tasks that could contribute to abnormal synergistic recruitment[51–53]. The interruption of direct corticospinal drive could enhance the influence of other indirect (i.e., reticulo- and vestibulospinal) pathways that produce less specific motor recruitment and lead to synergistic muscular activation in hemiparesis[46, 52]. Finally, changes in supraspinal influences due to stroke may also alter the regulation of spinal pathways involved in muscular coordination[54].

### Changes in heteronymous modulation after stroke

An increase in early heteronymous facilitation and a decrease in later inhibition of Sol voluntary EMG after FN stimulation were observed in the paretic leg of stroke individuals. Previous findings showed similar changes in heteronymous modulation using Sol H reflex[21]. Thus, changes in the excitability of propriospinal interneuronal pathways which integrate peripheral afferents do not only affect the reflex activity of motoneurones but also the activity triggered by descending voluntary drive. Early heteronymous facilitation and later inhibition are thought to be mediated by intersegmental group Ia afferents excitation and recurrent inhibition projecting from FN to Sol MNs, respectively, and moving through short propriospinal pathways[28, 29, 43, 55, 56]. Several spinal mechanism impairments have been reported after stroke and could contribute to modifications in heteronymous modulation. A reduction in presynaptic inhibition of group Ia terminals[57], a decrease in post-activation depression[22], an increase in group I and II intersegmental excitatory influences[58, 59] and changes in recurrent inhibition[60] are among the mechanisms that could potentially increase heteronymous facilitation and thus decrease later inhibition. Moreover, an increase in group II intersegmental excitatory influences has been reported during early stance in the gait cycle on the paretic side in stroke patients[61].

### Correlations between co-activation and heteronymous modulation

Some co-activation indexes correlated with the modified heteronymous modulations in the paretic leg. This demonstrates that the stroke patients who presented the highest levels of co-activations were also those who showed the highest levels of heteronymous facilitation. Although these correlations do not establish a causal relationship between the abnormal simultaneous activation of knee and ankle extensors during stance and changes in the heteronymous modulation, this finding opens the way for future studies to explore whether such a relationship exists. Most correlations were between reduced peak activation intervals and the impaired modulation. This raises the question as to whether changes in transmission of intersegmental pathways might contribute to reduce the activation delay between knee and ankle extensors during early stance. Changes in heteronymous pathways could theoretically contribute to co-activation of leg extensors in the paretic leg during the stance phase. This hypothesis is supported by the fact that the delay in intersegmental influence of Quads afferents on Sol motonoreures is short enough to affect the activity of ankle extensors in early stance. The central delay of the heteronymous modulation of Sol is only of 22 ms after FN stimulation and can last up to 40 ms in healthy subjects. In stroke participants, changes in the heteronymous modulation can be observed for up to 50 ms after the zero of central delay. Since EMG activity of knee and ankle extensors can overlap for 200 ms in the early stance phase[62], the facilitative heteronymous influence of the Quads has enough time to influence ankle extensors in this phase. The shortening of peak activation intervals in stroke patients is primarily due to early calf muscle activation, as opposed to changes in quadriceps activation timing[7]. One can therefore hypothesize that, in severely affected stroke individuals, activation of knee extensors at high levels at the beginning of the stance phase can produce an overall facilitative intersegmental influence on plantarflexors, which could contribute to triggering co-activation earlier during the mid-stance phase. Some evidence shows that the heteronymous modulation linking Quads and Sol is regulated according to postural tasks and gait phases[43, 63]. Changes in the regulation of this heteronymous modulation could be involved in coordination changes in these muscles in hemiparetic gait. Such changes in the regulation of heteronymous pathways during gait have been found in stroke patients[61, 64, 65] and might contribute to their functional deficits.

With respect to CAI, only the VL amplitude at Sol max significantly correlated with the heteronymous modulation. This might suggest that: 1) the intersegmental pathways assessed in the present study might be more relevant to the timing between knee and ankle extensors rather than the modulation of the intensity of co-activation, or 2) the effects of the quadriceps afferents on calf muscle EMG amplitude take time to build up and are seen after the quadriceps reach their maximal activation. Intersegmental influences occur between many muscles and some may be stronger than others. It is possible that the influences from calf muscles, when these muscles are stretched during eccentric contraction during mid-stance, could modulate the intensity of quadriceps activation in hemiparetic gait. Such modulation would involve other propriospinal pathways than those assessed in the present study. Intersegmental pathways projecting from Sol to Quads have been found in healthy humans[66, 67]. There are excitatory Ia afferents and recurrent inhibition influences[66, 67] but also facilitation from group II afferents projecting from Sol to Quads MNs[68]. Future studies should investigate whether potential changes in these other pathways could be related to increased amplitude of co-activation of knee extensors while plantarflexors are voluntarily activated in the paretic leg.

It is important to keep in mind that the correlations between co-activations and changes in heteronymous modulations found in the present study are not sufficient to establish a causal link between the presence of abnormal co-activation and the impairment of intersegmental mechanisms. In fact, several supraspinal and other spinal mechanisms can be affected by a stroke and contribute to the abnormal co-activation of knee and ankle extensors in hemiparetic gait. Abnormal co-activation and changes in intersegmental pathways observed in the present study might each be related to the extent of neurologic impairment after stroke, rather than being linked by a causal relationship. Moreover, some limitations of the study restrict the interpretation of the correlations between co-activation and heteronymous modulations. These limitations are related to the different conditions under which the assessments were performed. Heteronymous modulation was assessed under a static condition while participants were seated, whereas co-activation was assessed under a dynamic condition during gait. Although the conditions of assessment were different, correlations were found between the heteronymous modulation and co-activations, thereby suggesting a co-relationship between these measures. It can be hypothesized that the correlations found between the results of these assessments would have been greater had these assessments been performed under similar conditions. Future studies should investigate to what extent intersegmental pathways are modified during gait and whether changes in these pathways contribute to motor deficits observed in hemiparetic gait.

### Functional considerations

Our results showed that PAI_VL-Sol_ was correlated with levels of spasticity and motor impairment of the paretic leg. Furthermore, the level of co-activation of VL during peak activation of Sol (CAI_VL/Solmax_) was correlated with the levels of spasticity, motor impairment, and coordination of the paretic leg. It has been suggested that spasticity may account for early activation of calf muscles while these muscles are being stretched during the stance phase given the load on the limb[14]. This premature activation causes co-activation to occur because the quadriceps muscle is contracting. Results also suggest that the greater the amplitude of co-activation, the more uncoordinated the stroke subject is.

The correlation between co-activation indexes and motor impairment levels suggests that co-activation between VL and Sol could be either an adaptation (i.e., compensation) or a consequence of motor deficits of the paretic leg. It has been shown that deficits of selective muscular activation due to neurological impairment may be associated with global co-activation of knee and ankle extensors when severely affected stroke patients attempt to control weight bearing on the paretic leg during gait[8, 14, 69, 70]. Premature activation of ankle extensors during mid-stance may contribute to body weight support and compensate for knee extensor weakness in the stroke population. Activation of RF during mid-stance could also be an adaptation method to compensate for the lack of extension with the paretic leg and thus contribute to body weight support. Thus, increased co-activation of muscles acting at different joints could be a strategy to compensate for weakness at single joints. Such a strategy could be relevant to hemiparetic gait efficiency since strength measures at the hip[71, 72], knee[73, 74] and the ankle[75, 76] have been associated with gait performance after stroke.

Few studies have related abnormal co-activation with motor deficits after stroke. Co-activation of antagonist muscles at the ankle and synergistic co-activation of hip and knee extensors during alternate flexion and extension of the paretic leg in a supine position have been correlated with the level of motor impairment and gait performance[77]. It is still not clear whether abnormal synergistic co-activation observed during gait contributes to gait deficits or is an adaptation to compensate for these deficits. For example, inappropriate co-activation of leg flexors, including ankle dorsiflexors, could hamper propulsion provided by weak plantarflexors during stance[78] but assist with forward progression of the limb during the swing phase[77]. Similarly, co-activation of leg extensors could impede gait during the swing phase but contribute to weight bearing during the stance phase[77]. However, in the present study, no correlation was found between co-activation levels and walking performance measured by gait speed, despite variations from 0.5 to 1.3 m/s. This is consistent with some evidence demonstrating that improvement in gait speed is neither related to a reduction of abnormal co-activation patterns nor to a reduction in agonist-antagonists co-activation[79, 80]. Other factors, particularly adaptations of the other leg, must be considered when evaluating gait performance and gait speed after stroke.

Results of the present study have shown significant changes in the non-paretic leg. Hemiparetic participants spent more time in stance on the non-paretic leg compared to the paretic leg. The reduced percentage of time spent in stance on the paretic leg may be the consequence of weakness that prevents optimal weight acceptance on the affected side[81]. Gait speed is positively correlated with the percentage of time spent in the stance phase on the non-paretic side but not on the paretic side among stroke patients[81]. This suggests that changes in the percentage of time spent in stance and swing with the non-paretic leg may be relevant to hemiparetic gait as potential adaptations to compensate for motor deficits of the paretic leg. Evidence suggests that compensatory strategies of the non-paretic leg could be even more important than those of the paretic leg in hemiparetic gait performance[80].

## Conclusions

This study has quantified increased co-activation of knee and ankle extensors during the stance phase of gait in stroke individuals. PAI and CAI have been used to compare levels of temporal and relative amplitude of co-activation between stroke and healthy participants, respectively. Changes were identified with both co-activation indices after stroke. Moreover, temporal co-activation indexes correlated with changes in heteronymous modulations of soleus activity induced by femoral nerve stimulation. These results suggest that transmission changes in intersegmental pathways linking quadriceps to soleus could contribute to the timing of abnormal co-activation of knee and ankle extensors in hemiparetic gait. Further studies should investigate other propriospinal pathways, examine the extent to which co-activation of leg extensors on both the paretic and non-paretic side of stroke patients adapt to compensate for motor deficits, and determine whether this co-activation is the result of neurological impairments.

## Authors’ original submitted files for images

Below are the links to the authors’ original submitted files for images. Authors’ original file for figure 1 Authors’ original file for figure 2 Authors’ original file for figure 3 Authors’ original file for figure 4 Authors’ original file for figure 5 Authors’ original file for figure 6 Authors’ original file for figure 7
