# Supplementary figures and images for: Changes in activation timing of knee and ankle extensors during gait are related to changes in heteronymous spinal pathways after stroke
Source: J Neuroeng Rehabil. 2014 Oct 24;11:148. doi: 10.1186/1743-0003-11-148 (PMC4271343; doi:10.1186/1743-0003-11-148)

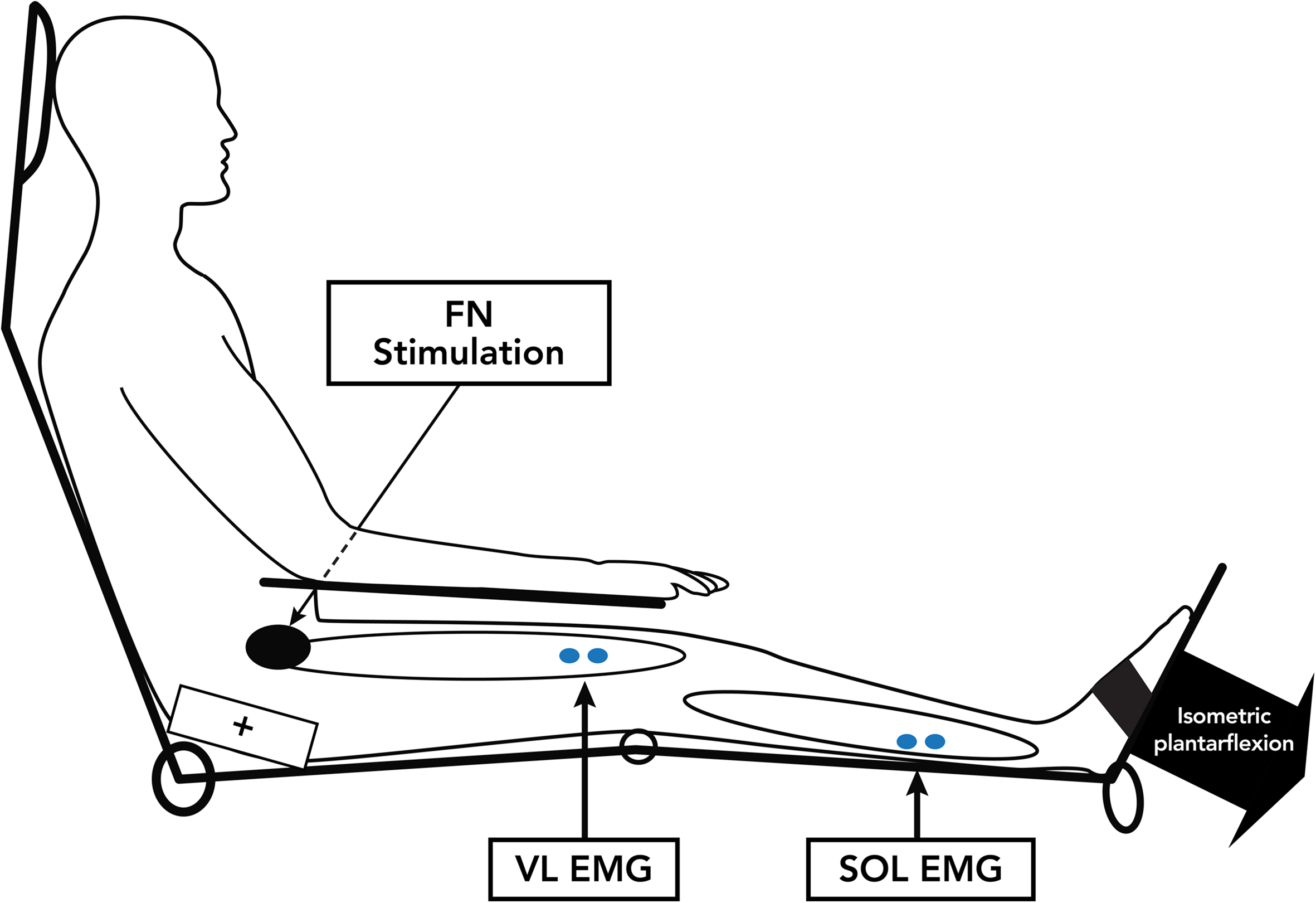

Supplement: Supplementary file 1 — Authors’ original file for figure 1 [file 12984_2014_679_MOESM1_ESM.tif]

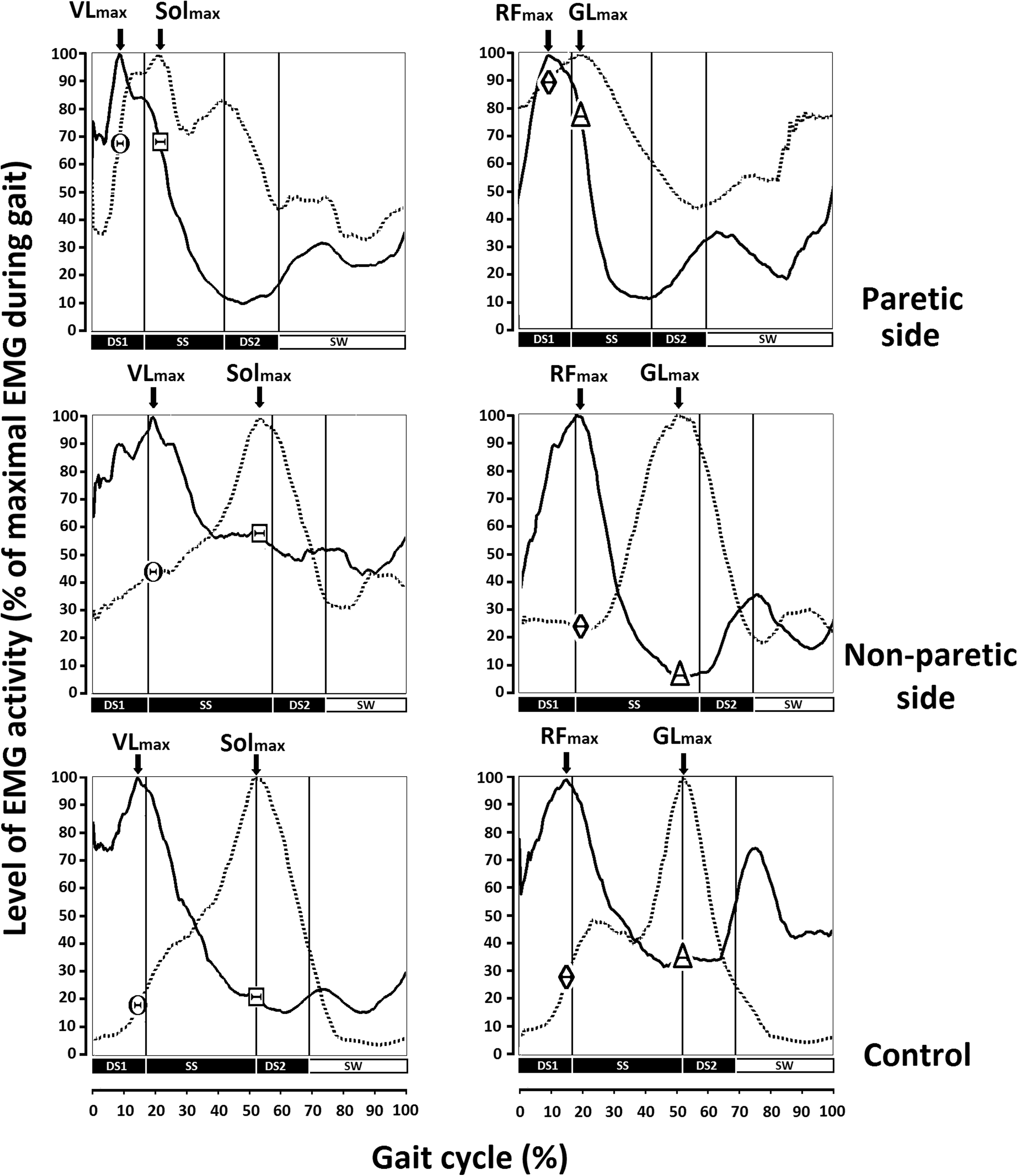

Supplement: Supplementary file 2 — Authors’ original file for figure 2 [file 12984_2014_679_MOESM2_ESM.tiff]

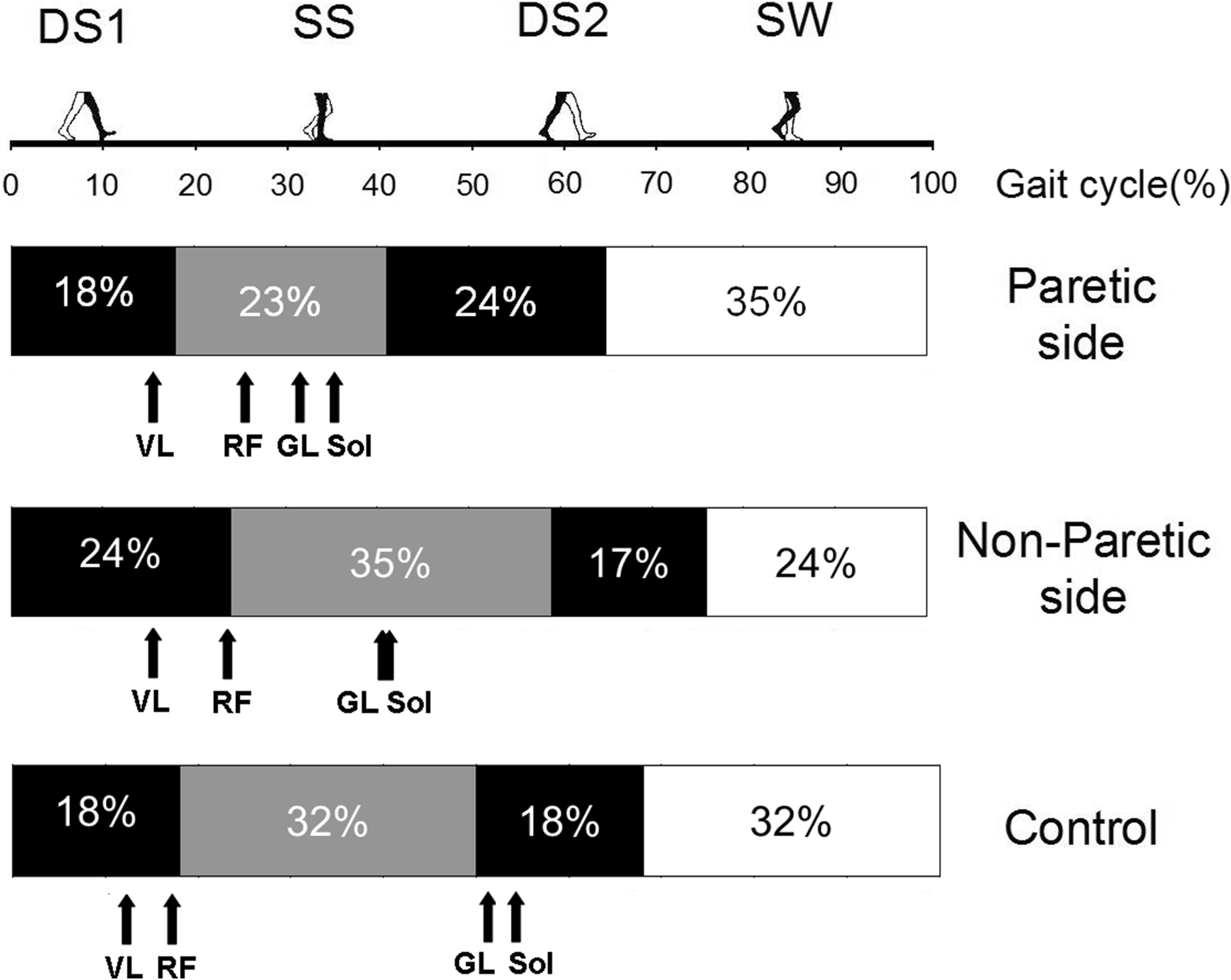

Supplement: Supplementary file 3 — Authors’ original file for figure 3 [file 12984_2014_679_MOESM3_ESM.tif]

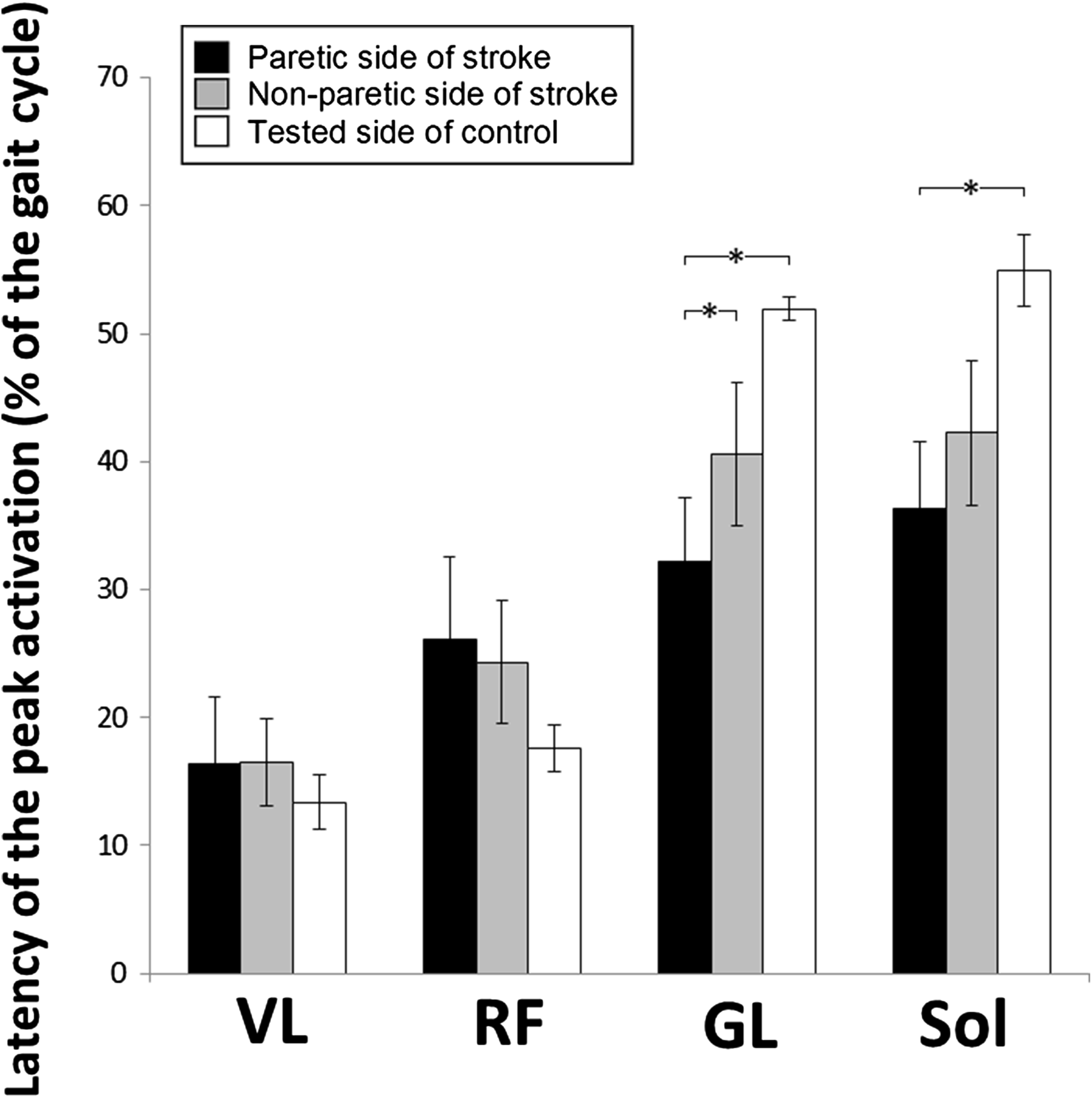

Supplement: Supplementary file 4 — Authors’ original file for figure 4 [file 12984_2014_679_MOESM4_ESM.tif]

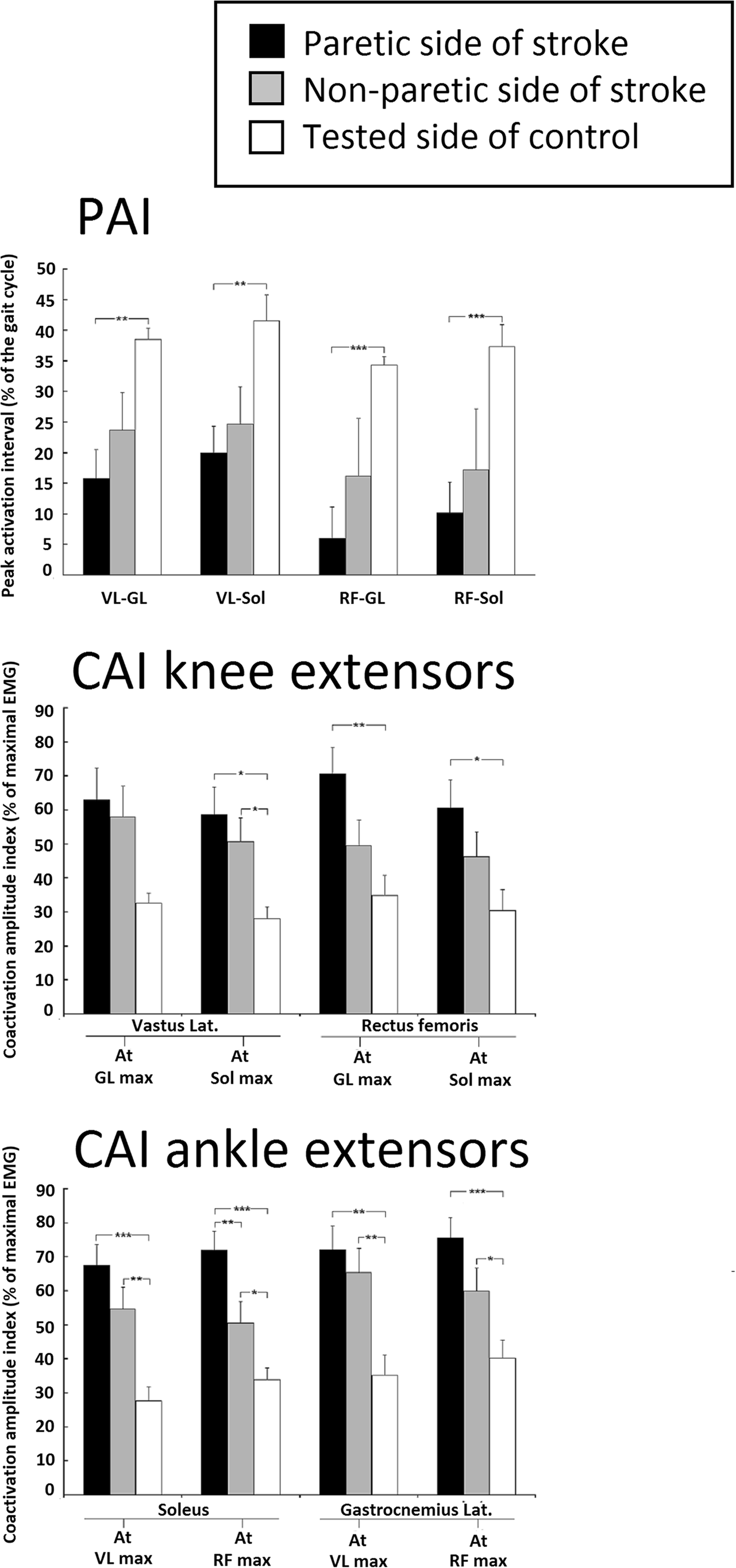

Supplement: Supplementary file 5 — Authors’ original file for figure 5 [file 12984_2014_679_MOESM5_ESM.tiff]

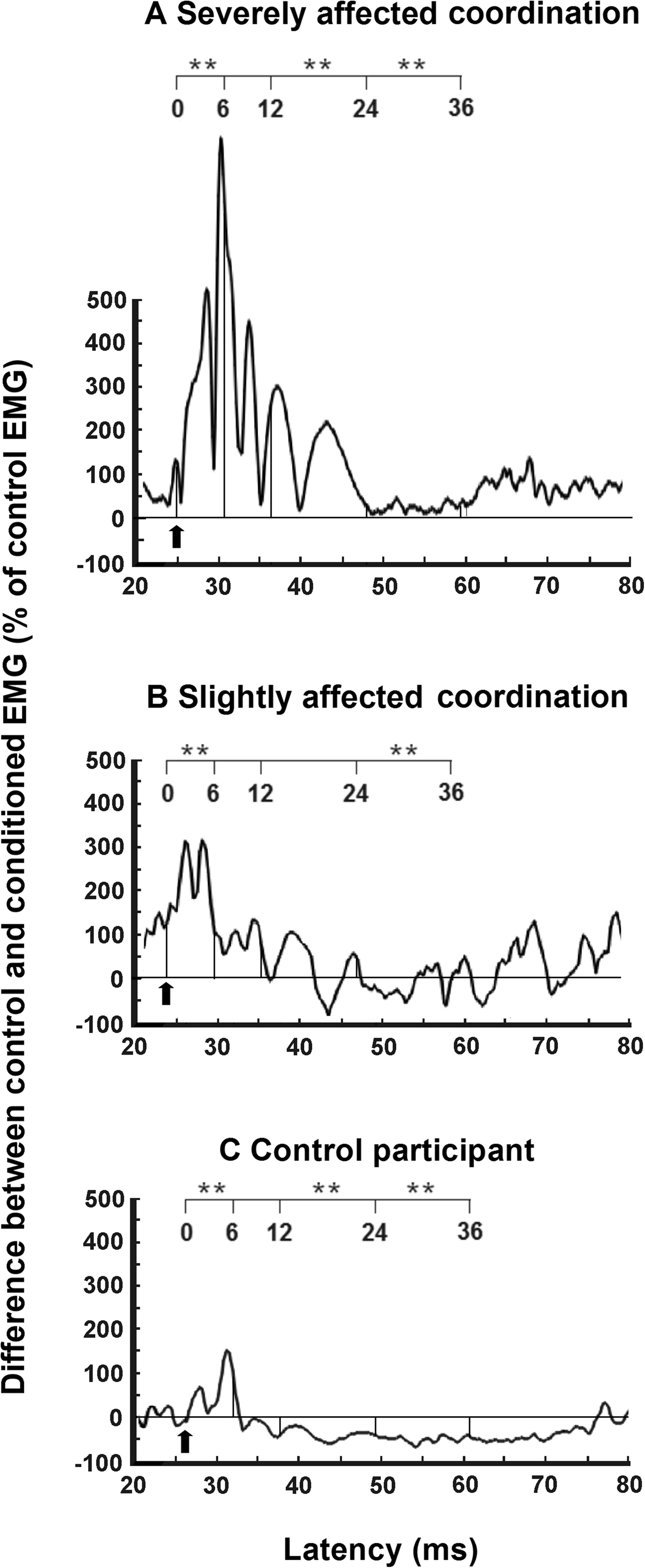

Supplement: Supplementary file 6 — Authors’ original file for figure 6 [file 12984_2014_679_MOESM6_ESM.tif]

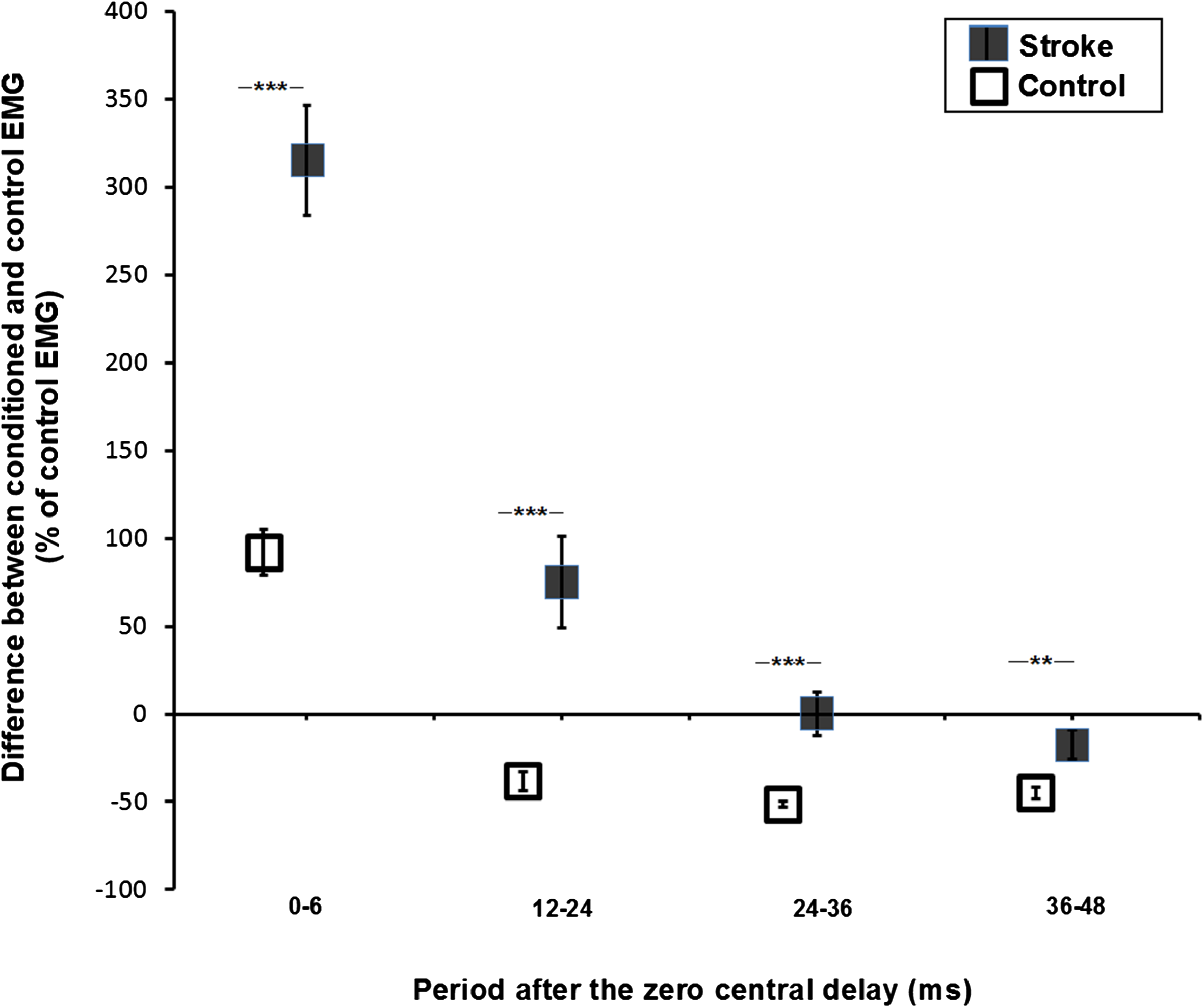

Supplement: Supplementary file 7 — Authors’ original file for figure 7 [file 12984_2014_679_MOESM7_ESM.tif]
